# HAND2-AS1 inhibits invasion and metastasis of cervical cancer cells via microRNA-330-5p-mediated LDOC1

**DOI:** 10.1186/s12935-019-1048-y

**Published:** 2019-12-27

**Authors:** Shengcai Chen, Jing Wang

**Affiliations:** Department of Gynaecology, Affiliated Hospital of Youjiang Medical University for Nationalities, No. 18, Zhongshan Second Road, Youjiang District, Baise, 533000 Guangxi People’s Republic of China

**Keywords:** Cervical cancer, HAND2 antisense RNA 1, MicroRNA-330-5p, Leucine zipper down-regulated in cancer 1, Lymph node metastasis, Proliferation

## Abstract

**Background:**

Cervical cancer is a serious disease with complicated pathogenesis and thus there is an urgent need to find novel targets for the treatment. Recently, long non-coding RNAs (lncRNAs) have emerged as critical factors in tumorigenesis. In this study, we aimed to investigate the mechanism of HAND2 antisense RNA 1 (HAND2-AS1) on the invasion and metastasis of cervical cancer cells.

**Methods:**

The expression patterns of HAND2-AS1, microRNA-330-5p (miR-330-5p) and leucine zipper down-regulated in cancer 1 (LDOC1) in cervical cancer were characterized by RT-qPCR and western blot analysis. Dual luciferase reporter assay and RIP were applied to verify relationship between HAND2-AS1, miR-330-5p and LDOC1. Fluorescence in situ hybridization (FISH) was used to detect the subcellular localization of HAND2-AS1. Besides, viability, invasion and migration ability of HeLa cells were investigated by cell counting kit-8 (CCK-8) and Transwell assays respectively. Hematoxylin–eosin staining was performed for lymph node metastasis detection. In addition, the tumor growth in nude mice was evaluated.

**Results:**

Low expression of HAND2-AS1 and LDOC1, and high expression of miR-330-5p were detected in cervical cancer tissues and cells. It was found that binding of HAND2-AS1 to miR-330-5p results in upregulation of LDOC1 expression. Also, overexpressed HAND2-AS1 and LDOC1 or down-regulated miR-330-5p inhibited expression of proliferation-associated proteins Ki-67, PCNA, migration-associated proteins N-cad and invasion-related proteins MMP-2, MMP-9 as well as lymph node metastasis. Moreover, HAND2-AS1 inhibited tumor formation and lymph node metastasis by binding to miR-330-5p in vivo.

**Conclusion:**

HAND2-AS1 promotes LDOC1 expression by competitively binding to miR-330-5p and consequently inhibiting cervical cancer cell invasion and metastasis. This could facilitate development of therapeutic strategies against cervical cancer.

## Background

Cervical cancer has become the fourth most commonly occurring cancer among women around the world [1]. In most cases, cervical cancer is caused by high-risk subtypes of the human papillomavirus (HPV) [2]. Recently, technologies like CryoPen and thermal coagulation have improved prevention and treatment of cervical cancer. Moreover, the effective screening has also greatly increased the rate of diagnosis of cervical cancer in patients [3]. In spite of the recent knowledge gained about the pathology of cervical cancer progression, further research demonstrating the tumorigenesis of cervical cancer is still necessary [4].

Long non-coding RNAs (lncRNAs) function as competing endogenous RNAs (ceRNAs). Thus, lncRNAs have an antagonistic effect on the post-transcriptional regulation of microRNAs (miRNAs or miRs) in gene expression and play a vital role during disease development [5]. LncRNAs have been reported to affect initiation and development of many cancers, including cervical cancer [6, 7] and can therefore provide insights for new targeted therapy against the progression of cervical cancer. LncRNA HAND2 antisense RNA 1 (HAND2-AS1) exhibits tumor suppressor activity in different types of cancer. A recent study has shown that HAND2-AS1 represses colorectal cancer progression by upregulating miR-1275-mediated KLF14 expression [8]. In cervical squamous cell carcinoma, HAND2-AS1 inhibits proliferation, migration and invasion abilities in both human papillomavirus (HPV)-positive and negative cells via downregulation of Rho-associated protein kinase 1 (ROCK1) [9]. Additionally, HAND2-AS1 was found to suppress cell migration and invasion while maintaining stem cell-like properties in non-small cell lung cancer through the interactions with TGF-beta1 [10]. Moreover, HAND2-AS1 facilitates the self-renewal of liver cancer stem cells by activating BMP signaling and consequently drives initiation of liver cancer [11]. The role of lncRNAs/miRNAs axis in cervical cancer has been documented in previous reports and thus could lead towards promising strategies for cervical cancer treatment [12, 13]. The ever-increasing evidence illustrates the important role played by miRNAs in multiple cancers, including cervical cancer. In cervical cancer, silencing miR-454-3p was able to promote cervical cancer cell apoptosis, thus inhibiting the cervical cancer [14]. Moreover, lncRNA WT1-AS inhibits the aggressiveness of cervical cancer cell via regulating p53 expression via sponging miR-330-5p [15]. All this evidence supports this study investigating the possible interplay between HAND2-AS1 and miR-330-5p in the development of cervical cancer.

## Materials and methods

### Ethics statement

The current study was performed with the approval of the Ethics Committee of Affiliated Hospital of Youjiang Medical University for Nationalities. Written informed consent was obtained from each participant. All animal experiments were approved by the Animal Ethics Committee of Affiliated Hospital of Youjiang Medical University for Nationalities and the principle of completing the experiment with the minimum number of animals and minimizing the pain inflicted upon the experimental animals strictly followed.

### Microarray-based analysis

Gene expression profiles in cervical cancer were obtained from GEO database. The “limma” package was used for differential gene expression analysis, with |log fold change| > 2 and *p* value < 0.05 set as threshold. The downstream miRNA targets of HAND2-AS1 were predicted using the RAID and RNA22 databases. Downstream target genes for miR-330-5p were predicted using the TargetScan (http://www.targetscan.org/vert_71/), miRDB (http://mirdb.org/miRDB/index.html), mirDIP (http://ophid.utoronto.ca/mirDIP/index.jsp#r), miRSearch (https://www.exiqon.com/miRSearch) and starBase databases (http://starbase.sysu.edu.cn).

### Study subjects

A total of 68 patients (aged 35–70 years with a mean age of 50.59 years) with cervical cancer who underwent surgery in the Department of Gynecology, at the Affiliated Hospital of Youjiang Medical University for Nationalities from April 2016 to April 2018 were included. Patients who were pregnant, breast-feeding or had other malignant tumors were excluded. There were 44 patients with the tumor size ≤ 4 cm and 24 patients with the tumor size > 4 cm. The 68 cases were categorized according to the International Clinical Obstetrics and Gynecology Union Clinical Staging Standard (2009 Edition) classification, including 22 cases in stage T1a, 16 cases in stage T1b, 22 cases in stage T2a and 8 cases in stage T2b. There were 21 cases with poorly differentiated tumor and 47 cases with moderately or highly differentiated tumor. Tumor tissues and adjacent tissues (> 5 cm from the edge of the tumor) were collected during the operation, which were immediately placed in liquid nitrogen for preservation. All specimens were confirmed by pathological examination, and no patients received chemotherapy or radiotherapy before surgery.

### Immunohistochemistry

The cervical cancer tissue sections were conventionally dewaxed by xylene and dehydrated by gradient alcohol. The sections were incubated in 3% hydrogen peroxide for 15 min, blocked with goat serum at 37 °C for 20 min and incubated with primary rabbit anti-leucine zipper down-regulated in cancer 1 (LDOC1) antibody (1:1000, ab86126, Abcam Inc., Cambridge, MA, USA) overnight at 4 °C. After a rinse with phosphate-buffered saline (PBS) for 15 min, the sections were incubated with the secondary goat anti-rabbit immunoglobulin G (IgG) (1:1000, ab150117, Abcam Inc., Cambridge, MA, USA) at 37 °C for 30 min, and washed with PBS for 15 min. Then, the sections were incubated in Strept avidin–biotin complex (SABC) (Boster Biological Engineering Co., Ltd., Wuhan, China) at 37 °C for 30 min, and stained with 3,3′-diaminobenzidine. Finally, the sections were stained with Hematoxylin for 1 min, destained with 1% hydrochloric acid alcohol, dehydrated, stained with aluminum carbonate for 30 s, and cleared in xylene for 15 min.

### Cell culture and transfection

Cervical cancer cell lines human cervical adenocarcinoma (HeLa) (3111C0001CCC000011) and Ca Ski (3111C0001CCC000101) cells were cultured with Roswell Park Memorial Institute (RPMI) 1640 medium (12633012, Shanghai Haoran Bio Technologies Co., Ltd., Shanghai, China). C-33A (3111C0001CCC000172) cells were cultured in the minimum essential medium (MEM) (12492-013, Shanghai Haoran Bio Technologies Co., Ltd., Shanghai, China) containing 10% fetal bovine serum. H1HeLa cells (3111C0001CCC000344) were cultured with Leibovitz medium (SNM541, Beijing Biolab Technology Co., Ltd., Beijing, China). All cells were from Cell Resource Center, Institute of Basic Medical Sciences, Chinese Academy of Medical Sciences. Normal human cervical epithelial cell lines (HUCEC) (BSC-00166804, ATCC, Manassas, VA, USA) were cultured in RPMI 1640 medium (12633012, Shanghai Haoran Bio Technologies Co., Ltd., Shanghai, China) containing 10% fetal bovine serum. All cells were cultured in a 37 °C incubator with an atmosphere of 5% CO_2_ in air. These cells were transfected with overexpression (oe)-HAND2-AS1, short hairpin RNA (sh)-HAND2-AS1, miR-330-5p mimic, miR-330-5p inhibitor, sh-LDOC1 or their corresponding controls. The above plasmids were purchased from Dharmacon (Lafayette, CO, USA).

### Dual luciferase reporter assay

The artificially synthetized HAND2-AS1-3′-untranslated region (3′-UTR) and LDOC1 3′UTR fragments were introduced into pMIR-reporter vector (Beijing Huayueyang Biotechnology Co., Ltd., Beijing, China) using endonuclease sites SpeI and Hind III. Mutation sites were designed on the complementary sequences of HAND2-AS1-wild type (WT) and LDOC1-WT respectively and the fragments were artificially synthesized. Using T4 DNA ligase, the target fragments were ligated to pMIR-reporter plasmids following restriction digestion. The luciferase reporter plasmids of WT and mutant (MUT) with correct sequence were co-transfected with mimic NC or miR-330-5p mimic into HEK-293T cells (Cell Resource Center in Shanghai Institute of Life Sciences, Chinese Academy of Sciences, Shanghai, China) respectively. Then, 48 h post transfection, cells were harvested and lysed to detect the luciferase activity using a luciferase assay kit (K801-200, BioVision, Milpitas, CA, USA) and Glomax 20/20 luminometer fluorescence detector (Promega, Madison, WI, USA).

### RNA binding protein immunoprecipitation (RIP)

RIP kit (Merck Millipore, Billerica, MA, USA) was applied to detect the binding of HAND2-AS1 and LDOC1 to Argonaute2 (Ago2) respectively. HeLa cells were lysed with radioimmunoprecipitation assay (RIPA) lysis buffer (P0013B, Beyotime Biotechnology Co., Shanghai, China) in an ice bath for 5 min and the lysate was then centrifuged at 14,000 rpm for 10 min at 4 °C to collect the supernatant. A part of the cell extract was taken out as an input, and a part was incubated with the antibody for coprecipitation. The magnetic bead-antibody complex was washed and resuspended in 900 μL RIP Wash Buffer and incubated with 100 μL cell extract at 4 °C overnight. The sample was placed on the magnetic pedestal to collect the magnetic bead-protein complex. The proteins in the samples and controls were then digested with proteinase K, and RNA was extracted for subsequent detection using polymerase chain reaction (PCR). The anti Ago2 antibody (ab32381, 1:50, Abcam Inc., Cambridge, MA, USA) was used for RIP and IgG (1:100, ab109489, Abcam Inc., Cambridge, MA, USA) was used as NC.

### Fluorescence in situ hybridization (FISH)

Subcellular localization of HAND2-AS1 in HeLa cells was analyzed by performing FISH as per the instructions of RiboTM lncRNA FISH Probe Mix (Red) (Guangzhou Ribobio Biotechnology Co., Ltd., Guangzhou, China). Briefly, 24-well plates were inoculated with 6 × 10^4^ cells/well. After overnight culture, the cells were then fixed, treated with 2 μg/mL protease K, glycine and acetylation reagent, and subsequently incubated with 250 μL prehybridization solution at 42 °C for 1 h. Next, the cells were incubated with 250 μL hybridization solution containing 300 ng/mL probe at 42 °C overnight. The next day, the cells were stained with 4′,6-diamidino-2-phenylindole (DAPI) staining solution diluted with PBS containing 0.1% Tween-20 (PBST) (Thermofisher scientific Co., Ltd., Massachusetts, USA) at a ratio of 1:800 for 5 min and then sealed with an anti-fluorescence quencher. Finally, cells in 5 different randomly selected fields of view were observed and photographed under a fluorescence microscope (Olympus, Tokyo, Japan).

### Transwell assay

The cell migration ability of cervical cancer cells was analyzed using a Transwell assay. Briefly, cancer cells were starved for 24 h. The next day, the cells were detached, centrifuged, resuspended into single cell suspension with cell density of 2 × 10^5^ cells/mL. Then 200 μL of this cell suspension was added to apical chamber, and 700 μL pre-cooled Dulbecco’s modified eagle’s medium was added into basolateral chamber. The cells were cultured in a 5% CO_2_ incubator at 37 °C. After 24 h, cells on the apical chamber and the basement membrane were wiped with a wet cotton swab, fixed, and stained with 0.1% crystal violet. Finally, the cells were observed and photographed under an inverted microscope. Five fields of view were randomly selected and the number of transmembrane cells was counted.

The cell invasion ability of cervical cancer cells was also analyzed. Briefly, the cells were harvested and resuspended into single cell suspension. The apical chamber coated with Matrigel gel (YB356234, Shanghai Yu Bo Biotech Co., Ltd., Shanghai, China) was added with 200 μL cell suspension, and 600 μL medium was added to the basolateral chamber. The chamber was incubated for 20–24 h at 37 °C. Then the cells on the upper surface were wiped with a cotton ball. The cells were then fixed, and stained with 0.1% crystal violet. The cells in 5 randomly selected fields of view were observed and counted under an inverted microscope (Leica, Wetzlar, Germany).

### Cell counting kit 8 (CCK-8)

HeLa cells (2 × 10^3^ cells/well) were seeded into a 96-well plate. After 24 h of transfection, 10 μL CCK-8 solution was added into each well at 0, 24, 48, 72, and 96 h respectively and the cells were incubated at 37 °C for another 4 h. Absorbance at 450 nm was measured using a microplate reader (Bio-Rad, Hercules, CA, USA). The ratio of the absorbance of the experimental group/control group was calculated, and the cell viability curve was drawn.

### Reverse transcription quantitative polymerase chain reaction (RT-qPCR)

Total RNA was extracted and reverse transcribed into complementary DNA (cDNA). RT-qPCR was performed using SYBR Premix EX Taq kit (RR420A, Takara Holdings Inc., Kyoto, Japan) in a real-time PCR instrument (ABI7500, ABI, Foster City, CA, USA). Three replicates were set for each sample. The primers were synthesized by Shanghai Biotech shown in (Table 1). U6 was used as an internal reference for miR-330-5p, while β-actin for HAND2-AS1 and LDOC1. The relative expression of genes was calculated using 2^−∆∆Ct^ method.

**Table 1:** Primer sequences for reverse transcription quantitative polymerase chain reaction

Primer	Sequence	
HAND2-AS1	Forward	5′-GGGTGTTTACGTAGACCAGAACC-3′
	Reverse	5′-CTTCCAAAAGCCTTCTGCCTTAG-3′
miR-330-5p	Forward	5′-CTTTGGCGATCACTGCCTCT-3′
	Reverse	5′-CTCTCTGCAGGCCGTGTG-3′
LDOC1	Forward	5′-GCTCCCCGAGTTTATCGTGC-3′
	Reverse	5′-TTCATGGCGTCGTTGCAGAA-3′
U6	Forward	5′-CGGAATTCCCCCAGTGGAAAGACGCG CAG-3′
	Reverse	5′-CGGTGTTTCGTCCTTTCCACAAG-3′
β-actin	Forward	5′-TCACCCACACTGTGCCCATCTACGA-3′
	Reverse	5′-CAGCGGAACCGCTCATTGCCAATGG-3′

*HAND2-AS1* HAND2 antisense RNA 1, *miR-330-5p* microRNA-330-5p, *LDOC1* Leucine zipper down-regulated in cancer 1

### Western blot analysis

Total protein was isolated from cells, separated by 10% polyacrylamide gel electrophoresis, and transferred to a polyvinylidene fluoride membrane. The membrane was then incubated at 4 °C overnight with rabbit anti-LDOC1 (1:1000, ab86126), rabbit anti-Ki-67 (1:1000, ab16667), rabbit anti-proliferating cell nuclear antigen (PCNA) (1:1000, ab92552), rabbit anti-N-cadherin (N-cad) (1:1000, ab18203), rabbit anti-E-cad (1:10,000, ab40772), rabbit anti-matrix metalloproteinases (MMP)-2 (1:1000, ab37150) and rabbit anti-MMP-9 (1:1000, ab73734). These primary antibodies were all from Abcam Inc. (Cambridge, MA, USA). After that, the membrane was washed with PBST. Subsequently, the membrane was incubated with horseradish peroxidase-labeled goat anti-rabbit IgG (1:10,000, ab6721, Abcam Inc., Cambridge, MA, USA) for 1 h, followed by scanning and development using an optical illuminometer (General Electric Company, Milwaukee, WI, USA). Finally, band intensities were quantified using Image Pro Plus 6.0 software (Media Cybernetics Inc., Maryland, USA). The relative expression of proteins was finally detected.

### Tumor formation in nude mice

A total of 18 specific pathogen-free (SPF) BALA/C nude female mice (age: 4–6 weeks, weight: 18–24 g) were acquired from Hunan SJA Laboratory Animal Co., Ltd. (Hunan, China). These mice were randomly divided into 3 groups with 6 mice in each group: oe-NC + NC mimic, oe-HAND2-AS1 + NC and oe-HAND2-AS1 + miR-330-5p mimic. The stably transfected cells were re-suspended in 10 mg/mL Matrigel (BD Biosciences, San Jose, CA, USA) at a ratio of 1:1, with cell density adjusted to 5 × 10^6^ cells/mL. Then 0.2 mL single cell suspension containing 1 × 10^6^ cells was injected subcutaneously into the left axilla of each mouse. Tumor size was observed and recorded every 3 days after 8 days of injection using a Vernier caliper, and tumor volume was calculated by the formula (mm^3^): tumor volume = length × width^2^ × 0.5. Then 30 days after tumor cell implantation, all nude mice were euthanized by cervical dislocation method and the tumor was removed and weighed.

### Hematoxylin–eosin (HE) staining

The paracancerous lymph nodes of the tumor tissue in nude mice were collected, fixed, embedded in paraffin and cut into 4-μm-thick sections. The sections were then dewaxed with xylene, hydrated with gradient ethanol, stained with Hematoxylin for 5 min, followed by soaking in tap water for 15 min or warm water (about 50 °C) for 5 min. After that, the sections were stained with eosin solution for 2 min followed by conventional dehydration, transparent and sealing with neutral resin. An inverted microscope (XSP-8CA, Shanghai Optical Instrument Factory, Shanghai, China) was used for photography and observation.

### Statistical analysis

All data were analyzed using SPSS 21.0 software (IBM Corp. Armonk, NY, USA). The measured values were expressed as mean ± standard deviation. Paired *t*-test was used for comparison between two groups, and Welch’s F test was performed for data correction. Data normality among multiple groups were analyzed by the Shapiro–Wilk method. The measurement data obeying normal distribution were analyzed by one-way analysis of variance (ANOVA). The Fisher’s least significant difference test was used to compare the average of the two groups and nonparametric Kruskal–Wallis test was used for data with unequal distribution. *p* < 0.05 was indicative of statistical significance.

## Results

### Low expression of HAND2-AS1 in cervical cancer and HeLa cells

**Fig. 1:**
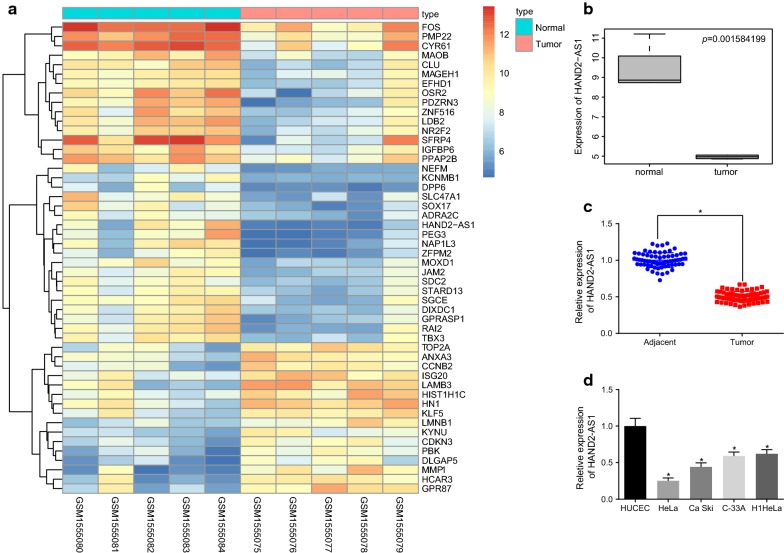
HAND2-AS1 is poorly expressed in cervical cancer and cells. **a** The heat map of some differentially expressed genes in the cervical cancer expression dataset GSE63678. The abscissa indicates the sample type and the ordinate indicates the gene expression level. The left dendrogram indicates the sample expression cluster, and the upper right histogram indicates color grade. **b** The expression of HAND2-AS1 in dataset GSE63678. The abscissa indicates the sample type, and the ordinate indicates the gene expression; the upper right indicates *p* value. **c** The expression of HAND2-AS1 in 68 cases of cervical cancer and adjacent tissues determined by RT-qPCR (N = 68), *p < 0.05 vs. the adjacent tissues. **d** The expression of HAND2-AS1 in human cervical cancer cell lines, *p < 0.05 vs. HUCEC cell line. All measurements were expressed by mean ± standard deviation. Paired *t*-test was used for comparison between cervical cancer tissues and adjacent tissues. The expression of each cell line was compared with one-way ANOVA, followed by Tukey’s post hoc test. The experiment was repeated three times. *HAND2-AS1* HAND2 antisense RNA 1, *RT-qPCR* reverse transcription quantitative polymerase chain reaction, *ANOVA* analysis of variance, *HUCEC* human cervical epithelial cell

Cervical cancer-related microarray dataset GSE63678 which included samples of both normal and cervical cancer, was obtained from the GEO database. Differential gene expression analysis revealed that 260 genes were differentially expressed in cervical cancer samples when compared to the normal samples (Fig. 1a). Among these differentially expressed genes, the level of HAND2-AS1 was significantly low in cervical cancer samples (Fig. 1b). RT-qPCR analysis of tissue samples obtained from 68 patients also showed that HAND2-AS1 was down-regulated in cervical cancer tissues when compared to adjacent tissues (Fig. 1c). Additionally, RT-qPCR analysis also revealed that HAND2-AS1 expression was much lower in cervical cancer cells when compared with HUCEC cells (all *p *< 0.05), with the lowest expression in HeLa cells (Fig. 1d). Therefore, HeLa cells were selected for subsequent studies.

### Overexpression of HAND2-AS1 inhibits proliferation and metastasis of cervical cancer cells

**Fig. 2:**
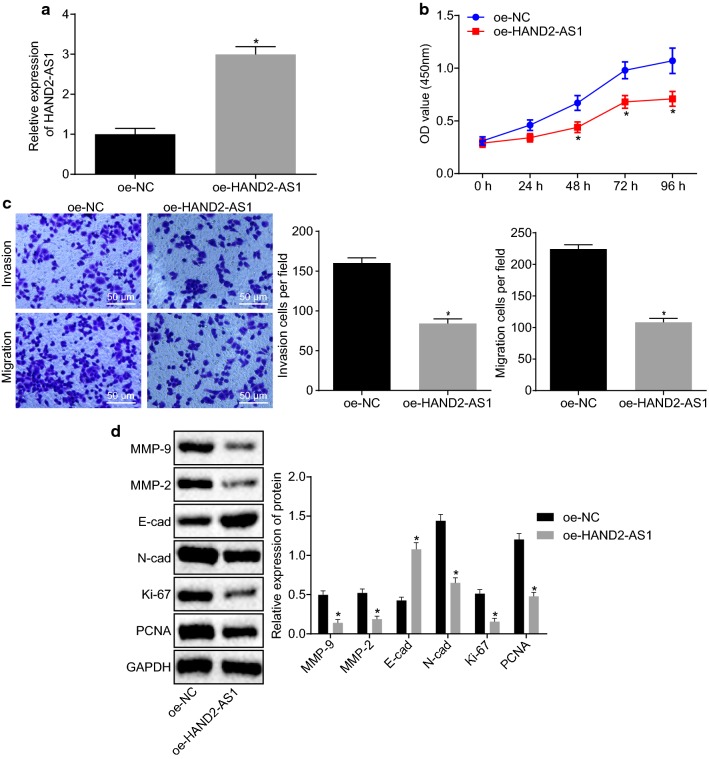
Overexpression of HAND2-AS1 slows down cervical cancer cell proliferation and metastasis. **a** oe-HAND2-AS1 and oe-NC transfection efficiency in HeLa cells tested by RT-qPCR. **b** HeLa cell viability detected by CCK-8 assay. **c** HeLa cell invasion and migration detected by Transwell assay. **d** The expression of proliferation-related proteins Ki-67, PCNA, migration-related proteins N-cad, E-cad and invasion-related proteins MMP-2 and MMP-9 in HeLa cells measured by Western blot analysis. *p < 0.05 vs. the cells treated with oe-NC. All measurements were expressed by mean ± standard deviation. Unpaired *t*-test was used for comparison between two groups. The experiment was repeated three times. *CCK-8* cell counting kit 8, *NC* negative control, *HeLa* human cervical adenocarcinoma, *PCNA* proliferating cell nuclear antigen, *MMP-2* matrix metalloproteinases 2, *MMP-9* matrix metalloproteinases 9, *N-cad* N-cadherin, *E-cad* E-cadherin

To test the effects of HAND2-AS1 on proliferation, invasion and migration of cells, HAND2-AS1 was overexpressed in HeLa cells followed by CCK-8 and Transwell assays. The transfection efficiency of HAND2-AS1 was confirmed by RT-qPCR (Fig. 2a) (*p *< 0.05). CCK-8 assay (Fig. 2b) and Transwell assay (Fig. 2c) revealed that overexpressed HAND2-AS1 reduced cell viability, invasion and migration ability of Hela cells. Moreover, western blot analysis of these cells revealed that expression of cell proliferation-related proteins Ki-67 and PCNA, migration-related protein N-cad, and invasion-related proteins MMP-2 and MMP-9 was down-regulated and expression of E-cad was elevated (Fig. 2d). These results indicate that overexpression of HAND2-AS1 in cervical cancer cells inhibits proliferation, invasion and metastasis.

### HAND2-AS1 binds to miR-330-5p

**Fig. 3:**
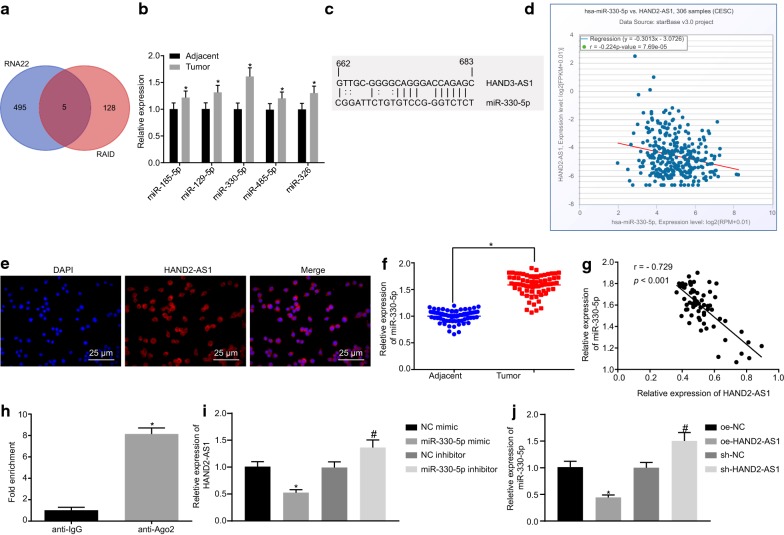
HAND2-AS1 targets miR-330-5p. **a** Prediction of downstream miRNA of HAND2-AS1. The two circles in the figure represent the results of prediction using RN22 and RAID database respectively, and the middle part represents the intersection of the two groups of data. **b** Detection of hsa-miR-185-5p, hsa-miR-129-5p, hsa-miR-330-5p, hsa-miR-485-5p and hsa-miR-326 expression in cancer tissues and adjacent tissues. N = 68. **c** Binding sites of miR-330-5p and HAND2-AS1 (N = 68, *p < 0.05 vs. adjacent tissues). **d** Correlation between HAND2-AS1 and miR-330-5p using TCGA. The abscissa indicates the expression of miRNA, and the ordinate indicates the expression of lncRNA. Each point represents the position of the expression of miRNA and lncRNA in the coordinates in each sample. **e** Subcellular localization of HAND2-AS1 (×400 magnification, scale bar was 25 μm). **f** Detection of miR-330-5p expression in 68 cases of cervical cancer and adjacent tissues by RT-qPCR (N = 68, *p < 0.05 vs. adjacent tissues). **g** Correlation analysis of HAND2-AS1 and miR-330-5p expression. **h** Binding of HAND2-AS1 and Ago2 determined by RIP. **i** HAND2-AS1 expression in HeLa cells transfected with miR-330-5p mimic or miR-330-5p inhibitor detected by RT-qPCR (*p < 0.05 vs. cells transfected with NC mimic; ^#^*p* < 0.05 vs. cells transfected with NC inhibitor). **j** miR-330-5p expression in HeLa cells transfected with oe-HAND2-AS1 or sh-HAND2-AS1 detected by RT-qPCR (*p < 0.05 vs. cells transfected with NC mimic; ^#^*p* < 0.05 vs. cells transfected with NC inhibitor). The above results were measurement data, and expressed as mean ± standard deviation. Paired *t*-test was applied for comparison of paired data obeying normal distribution and homogeneity of variance between two groups while unpaired *t*-test was used for comparisons of unpaired data between two groups. The experiment was conducted three times. *miR-330-5p* microRNA-330-5p, *Ago2* Argonaute2, *RIP* RNA binding protein immunoprecipitation, *TCGA* The Cancer Genome Atlas

In order to further understand the mechanism of action of HAND2-AS1 in cervical cancer, the downstream miRNAs of HAND2-AS1 were predicted using RNA22 and RAID database. Results obtained from both databases are shown in Fig. 3a along with the overlapping hits that revealed the following 5 potential downstream regulatory miRNAs of HAND2-AS1: hsa-miR-185-5p, hsa-miR-129-5p, hsa-miR-330-5p, hsa-miR-485-5p and hsa-miR-326. miR-330-5p expression was elevated in cervical cancer tissues following quantitative analysis (Fig. 3b).

As a next step to investigate the association between HAND2-AS1 and miR-330-5p, in silico analysis was done to identify the potential binding site of HAND2-AS1 in miR-330-5p (Fig. 3c). The Cancer Genome Atlas (TCGA) was used for correlation analysis revealing that HAND2-AS1 is negatively related to miR-330-5p (Fig. 3d). Besides, FISH indicated that HAND2-AS1 was mainly localized in the cytoplasm of HeLa cells (Fig. 3e). Besides, FISH indicated that HAND2-AS1 was mainly localized in the cytoplasm of HeLa cells (Fig. 3e). RT-qPCR results showed that miR-330-5p expression was much higher in cancer tissues than in adjacent tissues (Fig. 3f). Correlation analysis depicted in Fig. 3g illustrated an inverse correlation between expression of HAND2-AS1 and miR-330-5p in cancer tissue samples. The RIP showed that in HeLa cells, the level of HAND2-AS1 precipitated by anti-Ago2 antibody was remarkably higher in comparison to that by anti-IgG (all *p* < 0.05, Fig. 3h), indicating that HAND2-AS1 could form a complex with Ago2. Moreover, RT-qPCR displayed that HeLa cells transfected with miR-330-5p mimic had reduced expression of HAND2-AS1 while HeLa cells transfected with miR-330-5p inhibitor showed an elevated HAND2-AS1 expression (all *p* < 0.05, Fig. 3i). Furthermore, the levels of miR-330-5p expression in HeLa cells transfected with oe-HAND2-AS1 or sh-HAND2-AS1 were detected by RT-qPCR. Cells transfected with oe-HAND2-AS1 showed a decline in miR-330-5p expression whereas those with sh-HAND2-AS1 transfection an increase (all *p* < 0.05) (Fig. 3j). In conclusion, these results show that the complex of HAND2-AS1 and Ago2 could competitively bind to miR-330-5p and hence reduce the activity of miR-330-5p.

### HAND2-AS1 inhibits cervical cancer cell proliferation and metastasis via miR-330-5p

**Fig. 4:**
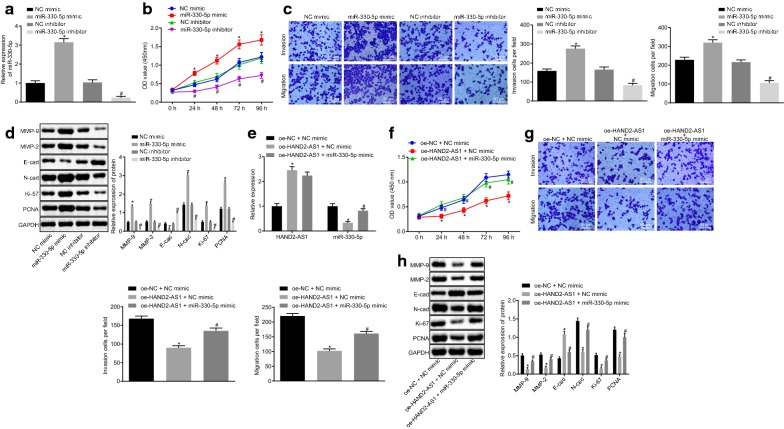
HAND2-AS1 mediates proliferation and metastasis of cervical cancer cells through miR-330-5p. **a** Transfection efficiency in HeLa cells tested by RT-qPCR. **b** HeLa cell viability detected by CCK-8 assay. **c** HeLa cell invasion and migration detected by Transwell assay. **d** The expression of proliferation-related proteins Ki-67, PCNA, migration-related proteins N-cad, E-cad and invasion-related proteins MMP-2 and MMP-9 in HeLa cells determined by Western blot analysis. *p < 0.05 vs. cells treated with NC mimic; ^#^*p* < 0.05 vs. cells transfected with NC inhibitor. **e** Transfection efficiency tested by RT-qPCR. **f** HeLa cell viability detected by CCK-8 assay. **g** HeLa cell invasion and migration detected by Transwell assay. **h** The expression of proliferation-related proteins Ki-67, PCNA, migration-related proteins N-cad, E-cad and invasion-related proteins MMP-2 and MMP-9 in HeLa cells determined by Western blot analysis. *p < 0.05 vs. cells treated with oe-NC and mimic NC; ^#^*p* < 0.05 vs. cells transfected with NC inhibitor or both oe-HAND2-AS1 and mimic NC. The data of each group were measurement data, expressed as mean ± standard deviation. One-way analysis of variance was used for comparison among multiple groups followed by Tukey’s post hoc test. The experiment was conducted three times. PCNA, proliferating cell nuclear antigen

The transfection efficiency of miR-330-5p was examined by RT-qPCR. The miR-330-5p expression was found to be reduced in cells treated with miR-330-5p inhibitor (all *p* < 0.05, Fig. 4a), indicating the successful transfection. Furthermore, treatment with miR-330-5p inhibitor was found to reduce cell viability, invasion and migration ability of HeLa cells, as well as inhibit expression of Ki-67, PCNA, N-cad, MMP-2 and MMP-9 while enhancing E-cad expression (all *p* < 0.05) (Fig. 4b–d).

It can be seen from the aforementioned results that HAND2-AS1 can competitively bind to miR-330-5p. HeLa cells were further transfected with oe-HAND2-AS1 or both oe-HAND2-AS1 and miR-330-5p mimic. The transfection efficiency was confirmed by RT-qPCR (Fig. 4e).

CCK-8 assay (Fig. 4f) and Transwell assay (Fig. 4g) showed that overexpressing HAND2-AS1 decreased viability, invasion and migration ability of HeLa cells (*p* < 0.05), which was reversed by miR-330-5p mimic (*p* < 0.05). Meanwhile, Western blot analysis (Fig. 4h) suggested that the protein expression of Ki-67, PCNA, N-cad, MMP-2 and MMP-9 was down-regulated while E-cad protein expression was increased in cells transfected with oe-HAND2-AS1 (*p* < 0.05), which was blocked by transfection of miR-330-5p mimic (*p* < 0.05). Taken together, these results suggest that HAND2-AS1 suppresses cervical cancer cell proliferation, invasion and metastasis via miR-330-5p.

### LDOC1 is a target gene of miR-330-5p

**Fig. 5:**
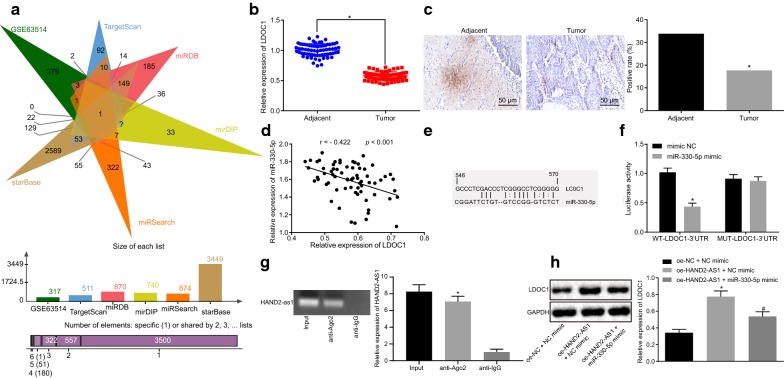
miR-330-5p targets and suppresses the expression of LDOC1. **a** Prediction of miR-330-5p downstream target genes; the 6 triangles represent the genes that were down-regulated in GSE63514, and the prediction results of target genes of miR-330-5p based on five different databases; the middle part represents the intersection of six sets of data. **b** Detection of LDOC1 expression in cancer tissues and adjacent tissues by RT-qPCR (N = 68, *p* < 0.05 vs. adjacent tissues). **c** Immunohistochemical detection of LDOC1 expression in cancer tissues and adjacent tissues (magnification: ×200, scale: 50 μm, N = 68, *p* < 0.05 vs. adjacent tissues). **d** correlation analysis of LDOC1 and miR-330-5p expression. N = 68. **e** Binding sites of miR-330-5p on LDOC1. **f** The binding of LDOC1 and miR-330-5p verified by dual luciferase reporter assay (*p < 0.05 vs. cells treated with mimic NC). **g** LDOC1 bound to Ago2 measured by RIP (*p < 0.05 vs. cells treated with anti-IgG). **h** LDOC1 protein expression in HeLa cells determined by Western blot analysis (*p < 0.05 vs. cells treated with oe-NC and NC mimic; ^#^*p* < 0.05 vs. cells transfected with oe-HAND2-AS1 and NC mimic). The paired *t* test was used to measure the data of cancer tissues and adjacent tissues. The positive rate of immunohistochemistry in the two groups was tested by Chi square test, while one-way analysis of variance was used for comparison among multiple groups followed by Tukey’s post hoc test. The experiment was performed three times. *LDOC1* leucine zipper down-regulated in cancer 1, *IgG* immunoglobulin G

In order to gain a deeper understanding of the downstream regulatory mechanism of miR-330-5p, the downstream target gene of miR-330-5p was predicted using databases such as TargetScan. Besides, the cervical cancer expression microarray dataset GSE63514 was utilized to find the significantly down-regulated genes. It was found that, there was only one gene, LDOC1 (Fig. 5a) that was common among the predicted target genes and those analyzed from GSE63514 dataset. Thus the levels of LDOC1 in patient samples were analyzed using RT-qPCR and immunohistochemistry (Fig. 5b, c) revealing the poor expression of LDOC1 in cervical cancer tissues in comparison to adjacent tissues (all *p* < 0.05).

Further, the correlation analysis of LDOC1 and miR-330-5p levels in tissue samples (Fig. 5d) showed that the expression between LDOC1 and miR-330-5p was negatively correlated. Besides, dual luciferase reporter assay suggested that (Fig. 5e, f) the luciferase activity of the Wt-LDOC1-3′-UTR in HEK-293T cells was down-regulated by miR-330-5p mimic (*p* < 0.05). RIP showed that level of LDOC1 precipitated by anti-Ago2 antibody in HeLa cells was higher compared with that precipitated by anti-IgG (*p* < 0.05), indicating that LDOC1 could form a complex with Ago2 (Fig. 5g). Moreover, western blot analysis revealed that expression of LDOC1 was significantly increased in HeLa cells upon transfection of oe-HAND2-AS1 (*p* < 0.05), which was blocked by co-transfection of oe-HAND2-AS1 and miR-330-5p mimic (*p* < 0.05, Fig. 5h). These results showed that miR-330-5p could target LDOC1 and inhibit the expression of LDOC1.

### miR-330-5p inhibits expression of LDOC1 to promote proliferation and metastasis of cervical cancer cells

**Fig. 6:**
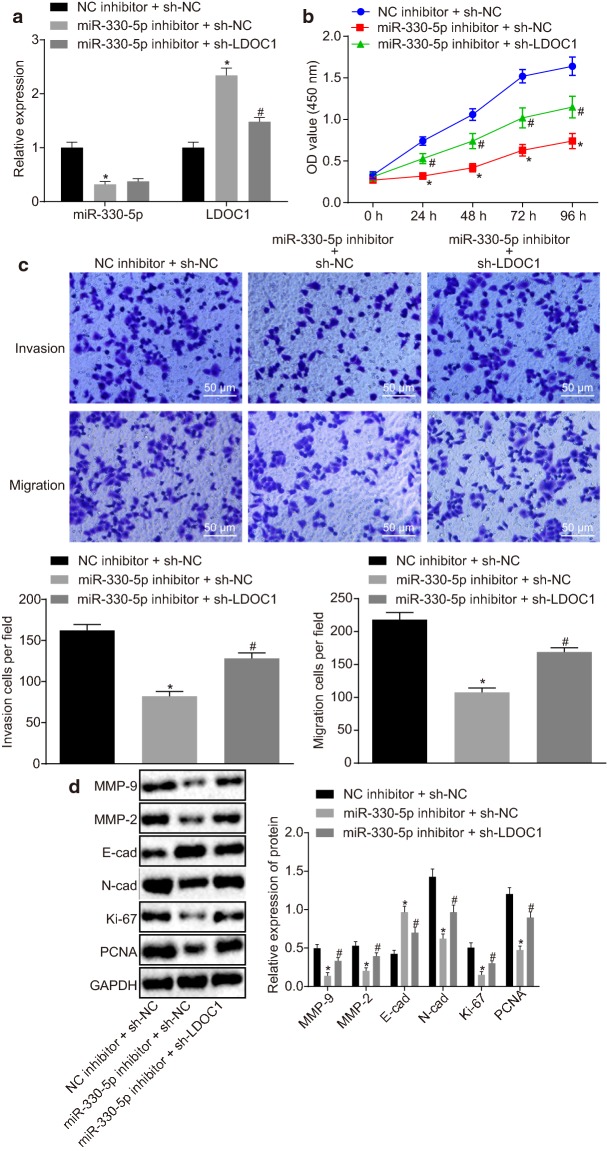
miR-330-5p down-regulates the expression of LDOC1 thus promoting proliferation and metastasis of cervical cancer cells. **a** Transfection efficiency in HeLa cells tested by RT-qPCR. **b** HeLa cell viability detected by CCK-8 assay. **c** HeLa cell invasion and migration detected by Transwell assay. **d** The expression of proliferation-related proteins Ki-67, PCNA, migration-related proteins N-cad, E-cad and invasion-related proteins MMP-2 and MMP-9 in HeLa cells determined by Western blot analysis. *p < 0.05 vs. cells treated with NC inhibitor and sh-NC; ^#^*p* < 0.05 vs. cells transfected with miR-330-5p inhibitor and sh-NC. The data of each group were measurement data, expressed as mean ± standard deviation. One-way analysis of variance was used for comparison among multiple groups followed by Tukey’s post hoc test. The experiment was conducted three times

The transfection efficiency was detected by RT-qPCR (Fig. 6a), which showed that miR-330-5p expression was decreased and LDOC1 expression was increased in the cells transfected with miR-330-5p inhibitor (*p* < 0.05). Also, compared with cells transfected with miR-330-5p inhibitor, miR-330-5p expression showed no significant difference (*p* > 0.05), and LDOC1 expression was decreased (*p *< 0.05) in the cells co-transfected with miR-330-5p inhibitor and sh-LDOC1, confirming the successful transfection.

CCK-8 assay was performed to detect cell viability (Fig. 6b), and cell invasion and migration were detected by Transwell assay (Fig. 6c). Cell viability, invasion and migration abilities were obviously reduced after miR-330-5p inhibitor treatment of cells (*p *< 0.05), which could be rescued by treatment with sh-LDOC1 (*p *< 0.05). Western blot analysis (Fig. 6d) displayed a decrease in the expression of Ki-67, PCNA, N-cad, MMP-2 and MMP-9 while an increase in expression of E-cad was observed in cells transfected with miR-330-5p inhibitor (*p *< 0.05), which could be reversed following LDOC1 silencing (all *p *< 0.05). These results indicate that miR-330-5p inhibits expression of LDOC1, thereby promoting proliferation and metastasis of cervical cancer cells.

### HAND2-AS1 inhibits expression of LDOC1 by binding to miR-330-5p to affect the proliferation and metastasis of cervical cancer cells

**Fig. 7:**
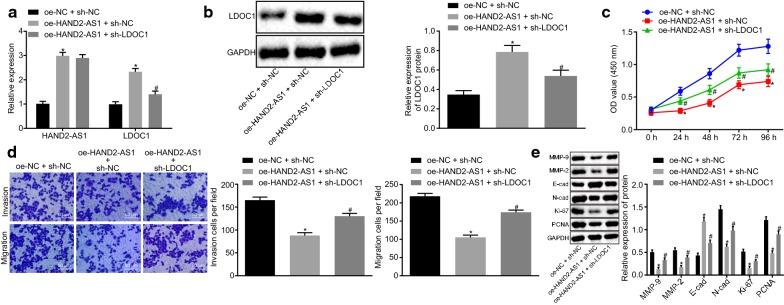
HAND2-AS1 regulates the expression of LDOC1 by binding to miR-330-5p to influence the proliferation and metastasis of cervical cancer cells. **a** transfection efficiency in HeLa cells tested by RT-qPCR. **b** LDOC1 protein expression in HeLa cells determined by Western blot analysis. **c** HeLa cell viability detected by CCK-8 assay. **d** HeLa cell invasion and migration detected by Transwell assay. **e** Western blot analysis of proliferation-related proteins Ki-67, PCNA, migration-related proteins N-cad, E-cad and invasion-related proteins MMP-2 and MMP-9 in HeLa cells. *p < 0.05 vs. cells treated with both oe-NC and sh-NC; ^#^*p* < 0.05 vs. cells co-transfected with oe-HAND2-AS1 and sh-NC. The data of each group were measurement data, expressed as mean ± standard deviation. One-way analysis of variance was used for comparison among multiple groups followed by Tukey’s post hoc test. The experiment was repeated three times

As a next step to understand the interplay of HAND2-AS1, miR-330-5p and LDOC1 in cervical cancer, HeLa cells transfected with appropriate plasmids were used to investigate cell proliferation, invasion and metastatic ability of cells. Again, the transfection efficiency was tested by RT-qPCR (Fig. 7a) and western blot (Fig. 7b). Expression of HAND2-AS1 and LDOC1 was increased in the cells transfected with oe-HAND2-AS1 (all *p *< 0.05). Compared with cells transfected with oe-HAND2-AS1, the expression of HAND2-AS1 did not change (*p* > 0.05), and the expression of LDOC1 was decreased (*p *< 0.05) in cell transfected with oe-HAND2-AS1-NC and sh-LDOC1 (Fig. 7a). Western blot analysis revealed that (Fig. 7b) LDOC1 expression in cells transfected with oe-HAND2-AS1 was increased (*p *< 0.05), while it was reduced in cells upon co-transfection of sh-LDOC1 (*p *< 0.05) confirming the successful transfection.

According to CCK-8 (Fig. 7c) and Transwell (Fig. 7d) assays, cell viability as well as invasion and migration ability was lowered after overexpression of HAND2-AS1 compared to cells treated with both oe-NC and sh-NC, which could be rescued by silencing LDOC1 (all *p *< 0.05). Western blot analysis (Fig. 7e) revealed that expression of Ki-67, PCNA, N-cad, MMP-2 and MMP-9 was decreased, while E-cad expression was increased in cells transfected with oe-HAND2-AS1 (all *p *< 0.05), which could be prevented after inhibiting LDOC1 (both *p *< 0.05). Taken together, these results confirm that HAND2-AS1 inhibits expression of LDOC1 by binding to miR-330-5p to affect the proliferation and metastasis of cervical cancer cells.

### In vivo study confirms that HAND2-AS1 inhibits tumorigenic ability and lymph node metastasis

**Fig. 8:**
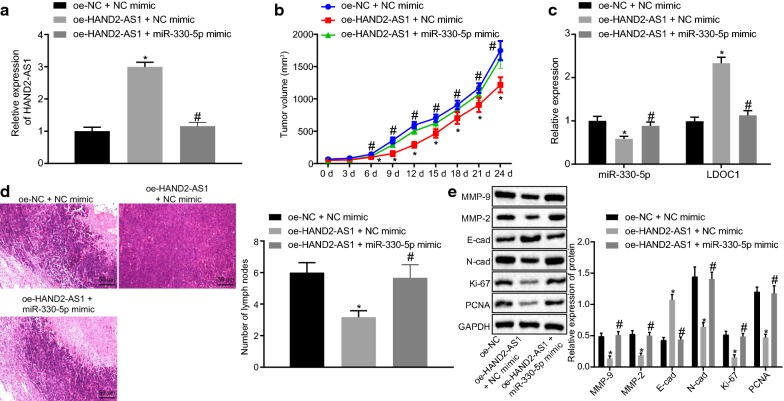
HAND2-AS1 inhibits tumorigenic ability and lymph node metastasis via binding to miR-330-5p in nude mice. **a** Expression of HAND2-AS1 in tumors from nude mice. **b** Tumorigenic ability of HeLa cells detected by tumor formation assay in nude mice. **c** miR-330-5p and LDOC1 expression in tumors determined by RT-qPCR. **d** Lymph node metastasis detected by HE staining (magnification: ×400; scale bar: 25 μm). **e** The expression of proliferation-related proteins Ki-67, PCNA, migration-related proteins N-cad, E-cad and invasion-related proteins MMP-2 and MMP-9 in tumors measured by Western blot analysis. *p < 0.05 vs. mice treated with oe-HAND2-AS1 + NC mimic. ^#^*p* < 0.05 vs. mice treated with oe-HAND2-AS1 + miR-330-5p mimic. The data were measurement data, expressed as mean ± standard deviation, N = 6. Unpaired *t* test was used for comparison between two groups. The experiment was performed three times. *HE* hematoxylin–eosin

RT-qPCR done to verify the overexpression of HAND2-AS1 in mice tumors (Fig. 8a) confirmed that expression of HAND2-AS1 was indeed increased in the tumor injected with cells expressing oe-HAND2-AS1 + NC mimic in comparison to oe-NC + NC mimic treatment (*p *< 0.05), suggesting successful transfection. However, expression of HAND2-AS1 was lowered in mice injected with cells expressing oe-HAND2-AS1 + miR-330-5p mimic than oe-HAND2-AS1 + NC mimic treatment (*p *< 0.05). Observation of tumor formation in nude mice (Fig. 8b) revealed that the tumor formation ability was weakened from the 9th day after injection with cells expressing oe-HAND2-AS1 + NC mimic in comparison to oe-NC + NC mimic treatment (all *p *< 0.05). By contrast, tumor formation ability was diminished in mice upon treatment with oe-HAND2-AS1 + miR-330-5p mimic than oe-HAND2-AS1 + NC mimic treatment (*p *< 0.05). Moreover, RT-qPCR (Fig. 8c) showed that expression of miR-330-5p was decreased while expression of LDOC1 was increased in nude mice injected with cells expressing oe-HAND2-AS1 + NC mimic compared to oe-NC + NC mimic treatment (all *p *< 0.05). A contrasting trend was detected in expression of miR-330-5p and LDOC1 in mice following treatment with oe-HAND2-AS1 + miR-330-5p mimic compared with oe-HAND2-AS1 + NC mimic treatment (*p *< 0.05). HE staining performed to detect lymph node metastasis in nude mice (Fig. 8d) suggested that the number of lymph nodes with metastasized cells was reduced in mice injected with cells expressing oe-HAND2-AS1 + NC mimic compared to oe-NC + NC mimic treatment (*p *< 0.05). Compared with mice treated with oe-HAND2-AS1 + NC mimic, there was an upward trend in the number of lymph nodes in mice following treatment with oe-HAND2-AS1 + miR-330-5p mimic (*p *< 0.05). Western blot analysis (Fig. 8e) exhibited that expression of Ki-67, PCNA, N-cad, MMP-2 and MMP-9 was decreased, while expression of E-cad was increased in tumors from mice injected with cells expressing oe-HAND2-AS1 + NC mimic when compared to mice injected with cells expressing oe-NC + NC mimic treatment (*p *< 0.05). An opposite trend was identified in expression of miR-330-5p and LDOC1 in mice following treatment with oe-HAND2-AS1 + miR-330-5p mimic compared with oe-HAND2-AS1 + NC mimic treatment (*p *< 0.05). These results showed that HAND2-AS1 could inhibit tumor formation and lymph node metastasis by binding to miR-330-5p in vivo.

## Discussion

Cervical cancer is known as a principal dangerous malignancy with huge threats to women across the world [4]. Although some diagnostics and treatment therapies including cervical cytology, pre-invasive disease management and HPV DNA testing applied in cervical cancer yield fruitful results, the prognosis of patients at advanced stage is often unfavorable. Consequently, further studies to enhance the prognosis are required [16]. This study investigated the role of HAND2-AS1 in cervical cancer, which uncovered that HAND2-AS1 could promote LDOC1 expression by competitively binding to miR-330-5p, thus inhibiting invasion and metastasis of cervical cancer cells.

We found that HAND2-AS1 was down-regulated in both cervical cancer tissues and cell lines, and overexpression of HAND2-AS1 inhibited the proliferation, migration and invasion of cervical cancer cells. The role of HAND2-AS1 has been investigated in a variety of cancers thus far. HAND2-AS1 is significantly down-regulated in endometrioid endometrial carcinoma tissues especially in poorly differentiated tumor tissues [17]. A recent study also revealed that HAND2-AS1 was down-regulated in the serum of patients with cervical squamous cell carcinoma and its overexpression could slow down cancer cell proliferation, migration and invasion [9]. Another study has revealed that HAND2-AS1 overexpression contributes to the reduction of cell invasion, migration as well as the decrease of proliferation in ovarian cancer [18]. Moreover, it was also indicated that HAND2-AS1 inhibited non-small cell lung cancer migration and invasion [10], which is partially consistent with our findings.

Recently, it has been found that HAND2-AS1 can exert a suppressive role in tumor progression by acting antagonistically with miRNAs [8, 19]. In the present study, we provided evidence that HAND2-AS1 could competitively bind to miR-330-5p and then reduce its expression. Furthermore, overexpressed HAND2-AS1 was shown to slow proliferation and metastasis of cervical cancer cells by down-regulating miR-330-5p. This finding is consistent with the current literature which has suggested that miRNA expression signatures can be a predictor for the progression of multiple cancers including breast cancer and cervical cancer [20]. In hepatocellular carcinoma, miR-330-5p was reported to be over-expressed at both cellular and tissue samples [21]. Moreover, it has been shown previously that overexpression of miR-330-5p promotes invasion and colony formation in cervical carcinoma cells [22]. In parallel with the above studies, our study revealed that inhibiting miR-330-5p attenuated the progression of cervical cancer. Furthermore, our findings verified that HAND2-AS1 inhibited tumorigenic ability and lymph node metastasis by binding to miR-330-5p in vivo. Another recent evidence also illustrates that increased expression of HAND2-AS1 inhibits in vivo tumor propagation of colorectal cancer [8]. Moreover, in past, loss of HAND2-AS1 has been correlated with the tumor grade, lymph node metastasis and recurrence of patients with endometrioid endometrial carcinoma [17].

LDOC1 is widely expressed in a variety of normal human tissues but is decreased in various human cancers. For instance, down-regulation of LDOC1 due to epigenetic silencing by promoter hypermethylation is widely known in oral, cervical and ovarian cancers [23]. Our RT-qPCR experimental results also demonstrated a poor expression of LDOC1 in cervical cancer tissues. Moreover, LDOC1 was identified to be a target gene of miR-330-5p. LDOC1 is a known miRNA target and has been previously validated as a target of hsa-miR-155 in glioblastoma. Here, LDOC1 is inhibited by hsa-miR-155 and ultimately drives cancer cell growth [24]. Likewise, in the present study we observed that miR-330-5p inhibits the expression of LDOC1 and thus promotes proliferation, invasion and metastasis of cervical cancer cells. More interestingly, in agreement with our findings, another group found that LDOC1 was poorly expressed in cervical cancer, and overexpression of LDOC1 was able to induce cell apoptosis in cervical cancer cells [25].

Another key finding was that overexpression of HAND2-AS1 inhibited proliferation, migration and invasion of cervical cancer cells through upregulating LDOC1 by binding to miR-330-5p. A growing number of studies have flagged a crosstalk among lncRNAs, mRNAs and shared miRNAs along with their targets in several cancers [26, 27]. For instance, the network of lncRNA-miRNA-mRNA interaction may have the potential to be used to refine biomarker predictions for developing novel therapeutic approaches in breast cancer [28]. HOTAIR functions as a ceRNA to regulate notch3 expression via miR-613 in pancreatic cancer [29]. Likewise, lncRNA SNHG12 was found to upregulate SIRT1 by targeting miR-199a, consequently restarting cerebral ischemia/reperfusion injury [30].

## Conclusions

**Fig. 9:**
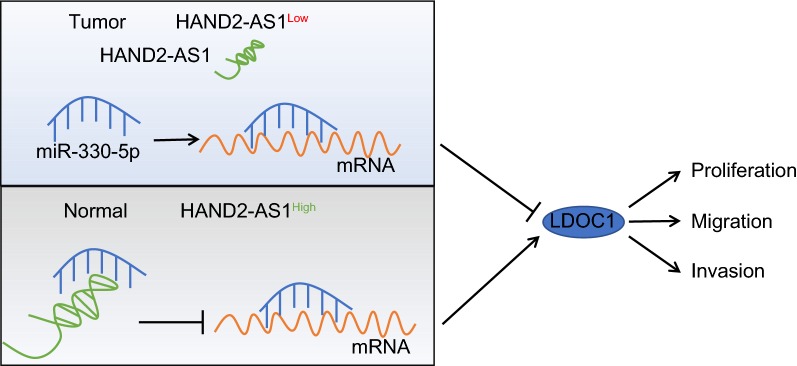
A mechanism map depicting the role of HAND2-AS1 in cervical cancer. HAND2-AS1 is under-expressed in cervical cancer. HAND2-AS1 promotes LDOC1 expression by competitively binding to miR-330-5p, thereby inhibiting invasion and metastasis of cervical cancer cells

To sum up, HAND2-AS1 promotes LDOC1 expression by competitively binding to miR-330-5p, thereby inhibiting invasion and metastasis of cervical cancer cells (Fig. 9). Therefore, HAND2-AS1 may be valuable as a new biomarker for prognosis and a promising therapeutic target for patients with cervical cancer. Nonetheless, the current study only presents the theoretical basis of this mechanism in the HAND2-AS1/miR-330-5p/LDOC1 axis. Therefore, detailed pre-clinical and clinical studies with fully developed anti-cancer therapeutic agent are required.

## Data Availability

The datasets generated/analyzed during the current study are available.

## Abbreviations

HAND2-AS1: HAND2 antisense RNA 1
miR-330-5p: microRNA-330-5p
LDOC1: leucine zipper down-regulated in cancer 1
FISH: fluorescence in situ hybridization
HE: hematoxylin–eosin
HPV: human papillomavirus
ceRNAs: competing endogenous RNAs
lncRNAs: long non-coding RNAs
IgG: immunoglobulin G
SABC: Strept avidin–biotin complex
MEM: minimum essential medium
oe: overexpression
sh: short hairpin RNA
3′-UTR: 3′-untranslated region
WT: wild type
MUT: mutant
RIPA: radioimmunoprecipitation assay
PBS: phosphate-buffered saline
RPMI: Roswell Park Memorial Institute
Ago2: argonaute2
FISH: fluorescence in situ hybridization
DAPI: 4′,6-diamidino-2-phenylindole
RIP: RNA binding protein immunoprecipitation
RT-qPCR: reverse transcription quantitative polymerase chain reaction
cDNA: complementary DNA
PCNA: proliferating cell nuclear antigen
N-cad: N-cadherin
MMP: matrix metalloproteinases
CCK-8: cell counting kit 8
SPF: specific pathogen-free
PBST: phosphate-buffered saline containing 0.1% Tween-20
ANOVA: analysis of variance
